# Salivary Microbiome and Cigarette Smoking: A First of Its Kind Investigation in Jordan

**DOI:** 10.3390/ijerph17010256

**Published:** 2019-12-30

**Authors:** Walid Al-Zyoud, Rima Hajjo, Ahmed Abu-Siniyeh, Sarah Hajjaj

**Affiliations:** 1 Department of Biomedical Engineering, School of Applied Medical Sciences, German Jordanian University, Amman 11180, Jordan; sara.j.hjj@gmail.com; 2 Department of Pharmaceutical Sciences, School of Pharmacy, Al-Zaytoonah University of Jordan, Amman 11733, Jordan; rih727@g.harvard.edu; 3 Clinical Laboratory Sciences Department, College of Applied Medical Science, Taif University, Taif 11099, Saudi Arabia; akabusiniyeh@tu.edu.sa

**Keywords:** saliva, microbiome, microbiota, smoking, 16S rRNA, next-generation sequencing, operational taxonomic unit (OTU), bioinformatics, Jordan

## Abstract

There is accumulating evidence in the biomedical literature suggesting the role of smoking in increasing the risk of oral diseases including some oral cancers. Smoking alters microbial attributes of the oral cavity by decreasing the commensal microbial population and increasing the pathogenic microbes. This study aims to investigate the shift in the salivary microbiota between smokers and non-smokers in Jordan. Our methods relied on high-throughput next-generation sequencing (NGS) experiments for V3-V4 hypervariable regions of the 16S rRNA gene, followed by comprehensive bioinformatics analysis including advanced multidimensional data visualization methods and statistical analysis approaches. Six genera—*Streptococcus*, *Prevotella*, *Vellionella*, *Rothia*, *Neisseria*, and *Haemophilus*—predominated the salivary microbiota of all samples with different percentages suggesting the possibility for the salivary microbiome to restored after quitting smoking. Three genera—*Streptococcus*, *Prevotella*, and *Veillonella*—showed significantly elevated levels among smokers at the expense of *Neisseria* in non-smokers. In conclusion, smoking has a definite impact on shifting the salivary microbiota in smokers. We can suggest that there is microbial signature at the genera level that can be used to classify smokers and non-smokers by Linear Discriminant Analysis Effect Size (LEfSe) based on the salivary abundance of genera. Proteomics and metabolomics studies are highly recommended to fully understand the effect of bacterial endotoxin release and xenobiotic metabolism on the bacterial interrelationships in the salivary microbiome and how they affect the growth of each other in the saliva of smokers.

## 1. Introduction

One of the foremost public health problems affecting the world today is smoking, which represents a preventable cause of premature death [1]. Smoking has a crucial implication in causing many common diseases such as cancer, chronic obstructive pulmonary diseases, and periodontitis [2,3,4]. The impact of smoking tobacco on periodontal health is not a novel concept; however, several studies have shown the significant adverse effect of smoking on the microbiome and cytokines expression of the buccal mucosa [5]. Nevertheless, tobacco in addition to other environmental factors may affect the equilibrium of the oral microbiome by inducing a possible alteration in functional pathways and allowing oral pathogens to grow, which ultimately leads to several diseases [6,7,8]. Furthermore, different oral pathogens harm the development and function of innate and adaptive immunity of the host [9]. The ease of saliva collection indicates that saliva microbiome studies have a vital implication in disease diagnosis or prognosis [10]. Jordan is one of the many countries dealing with the smoking epidemic. A cross-sectional survey was conducted among 11 to 18-year-old school students, including boys and girls, from an important governorate in Jordan. The study surprisingly showed a high rate of smoking and particularly dual tobacco consumption, cigarettes, and waterpipe [11]. The studies emphasized that there is an increasing smoking rate among the Jordanian population, especially among the youth, which indicates awareness insufficiency of the destructive effects of smoking and the necessity for prevention programs to handle such knowledge deficiency. Therefore, this study is to investigate the impact of smoking on oral cavity microbiome components among adult Jordanian smokers and the comparison between smoking and non-smoking subjects by using high-throughput 16S rRNA gene sequencing via next-generation sequencing technology (NGS). The outcome of the current study is expected to help identify the interactions between smoking and the salivary microbiome.

The oral microbiome is an essential player that induces a dynamic equilibrium with the immune-inflammatory response of the host [12]. The human oral cavity is one of the entries of the respiratory tract, and the main entry point for several microorganisms, primarily airborne pathogens, and those transferred through saliva. The salivary microbiome possesses its characteristic microorganisms and interacts with other microbiomes in the human body, especially that of the intestinal tract [13]. There is excellent genera diversity in the human salivary microbiome; therefore, it is essential to understand the role of these known and unknown genera in the oral cavity and how they interact with the microbiomes of other systems in the human body [13].

The precision in determining the oral microbiome is not easily attainable because the oral cavity is an open system exposed continuously to bacteria present in food and water in addition to bacteria contracted through social contact. It is challenging to determine whether existing colonizations are a long-term diversity or not. A diversity that makes this community able to provide an appropriate response to each environmental stress or factors such as smoking, diet, oral hygiene, and drug consumption, e.g., antibiotics. Tobacco smoking generates carcinogens that contain distinct nitrosamines and free radicals capable of inhibiting antioxidant enzymes. In turn, an inhibited antioxidant enzyme makes the oral epithelial cells unprotected against the damaging effects of thiocyanate ions and hydroxyl free radicals. Thiocyanate ions and free radicals could react with DNA, adversely, and therefore, open the gateway to the progression of oral cancer [14]. By inhibiting granulocyte function, smoking impairs host defenses and affects the immune system [15].

Furthermore, subsequent nicotine metabolites trigger vasoconstriction and prejudice the role of polymorphonuclear cells and macrophages as well as decrease the number of lymphocytes, which may adversely affect the production of B-cells and antibodies [16]. Furthermore, smoking contributes to the increase in the number of neutrophils in peripheral blood [17]. The changes that occur as a result of activating inflammatory cells, which leads to the release of free radicals, were found to influence a move to malignancy by lipids’ peroxidation or DNA damage. The phyla *Firmicutes*, *Bacteroidetes*, *Proteobacteria*, *Actinobacteria*, *Spirochaetes*, and *Fusobacteria* dominate the oral cavity, accounting for more than 95% of the species [18]. Various health-associated bacteria have been known to be antagonistic to oral pathogens; *Streptococcus salivarius* strain K12, for example, produces a bacteriocin that prevents the growth of Gram-negative species linked to periodontitis [19].

## 2. Materials and Methods

### 2.1. Study Subjects

One hundred (n = 100) human subjects participated in this study; 57 were males and 43 were females. According to the smoking status, 51 were non-smokers and 49 were smokers. The inclusion criteria required that all human subjects were antibiotic-free for the last three months preceding the study by ensuring that no one has consumed antibiotics in that period. Inclusion criteria for smokers required that all smoker subjects smoked at least one cigarette per day. The exclusion criteria, on the other hand, required the rejection of human subjects who had a history of any chronic oral diseases.

Additionally, saliva collection from all subjects was taken half an hour before, or an hour after eating. Signed informed consent and answered questions were obtained from all participants in this study according to the declaration of Helsinki. The Council of Scientific Research at the German Jordanian University has approved the proposal of the study based on decision #31/3/2016 as stated in letter #389/6/4/10.

### 2.2. Sample Collection, Processing, and Storage

All human subjects had to spit their unstimulated saliva into the OMNIgene•ORAL OM-501™ funnel, which is commercially available by DNA Genotek, ON, Canada. Subjects kept on spitting until the amount of spat liquid, excluding bubbles, reached the filled line mark indicated on the wall of the collecting tube. All human subjects were required to hold the collecting tubes upright with one hand and close the funnel lid with the other hand. A liquid DNA stabilizer, placed in the tube cover, was automatically released at this stage into the tube after replacing the funnel with the tube cap to firmly close the collecting tube. The DNA stabilizer stabilizes the microbial DNA in saliva for up to one year at room temperature. The DNA stabilizer was then mixed with the collected liquid sample for 10 s. The samples were shipped at room temperature to DNA Genotek GenoFIND Services, Norcross, GA, USA, for complete processing.

### 2.3. DNA Extraction and Quality Controls

A 250 µL aliquot of each sample was extracted using MO BIO’s PowerMag™ microbial DNA isolation kit (27200-4) (MO BIO Laboratories Inc., Carlsbad, CA, USA) optimized on the KingFisher automated extraction platform. A proprietary bead-beating step with glass beads and a plate shaker was used to maximize recovery of DNA from low-abundance and challenging to lyse organisms. The concentration of extracted DNA was determined by Qubit measurement, and an estimate of sample purity was determined with spectrophotometry by measuring the A260/A280 absorbance ratio. Quality control checks are tabulated in Appendix A data (Table A1).

### 2.4. DNA Sequencing

Illumina sequencing adapters and dual-index barcodes (Nextera XT indices) were added to the amplicon target via polymerase chain reaction (PCR) amplification. Samples were run on Bioanalyzer, spot-checking for amplicon size. The 16S sequencing (2 × 300 bp PE V3-V4) was performed on Illumina’s MiSeq platform (Illumina Inc., San Diego, CA, USA). Paired-end reads from each sample were merged, screened for length, and filtered for quality using DNA Genotek’s proprietary 16S pre-processing workflow. The sequence data were submitted to NCBI *BioProject* under accession number PRJNA579773.

### 2.5. Taxonomic Classification

High-quality sequences were aligned to the curated reference database at 97% similarity using the NINJA-OPS algorithm, version 1.5.1 [20]. At 97% sequence identity, each operational taxonomic unit (OTU) represents a genetically unique group of biological organisms. These OTUs were then assigned a curated taxonomic label based on the SILVA taxonomic database, version 123 [21]. The relative abundance of all taxa at the phylum and genus levels were plotted to visualize broad taxonomic differences between individual samples and between sample groups. Genera found at <1% mean abundance across samples were grouped as “other” for visualization purposes. MicrobiomeAnalyst, a web-based data analysis tool, was chosen to perform Univariate statistical analysis for features at the phyla and genera levels; features were considered significant based on their adjusted cut-off ≤ 0.05. This web-based tool has been reported and is currently hosted by the Xia lab at McGill University, QC, Canada [22].

### 2.6. Rarefaction

**Figure A1:**
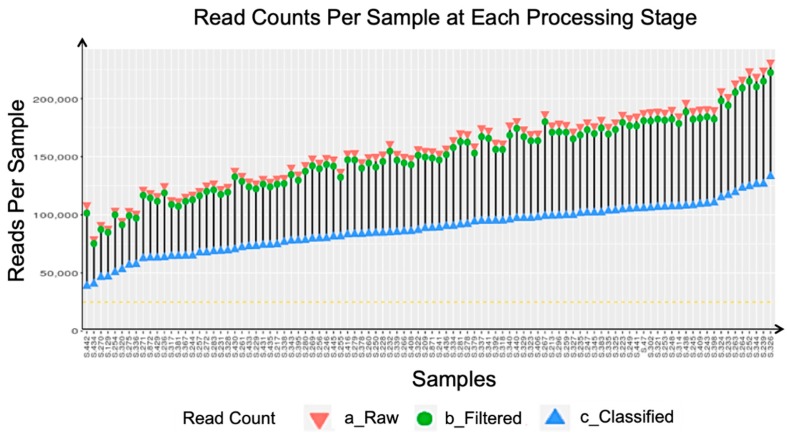
Sequence counts per sample for raw, filtered, and classified sequences, ordered by increasing classified sequence counts. The dashed gold line indicates the 25,000 sequence threshold used for rarefaction.

**Figure A2:**
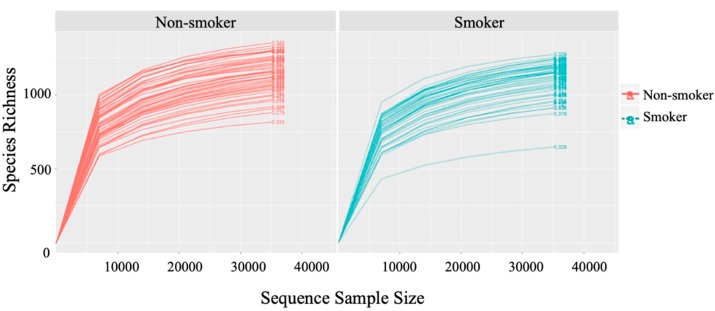
Rarefication curve using filtered dataset.

All samples were rarefied after taxonomic classification. The cutoff for rarefaction was set at 25,000 classified sequences per sample. However, no sample had less than 25,000 classified sequences, thus, all samples were included in the downstream analysis (see Figure A1 and Figure A2).

### 2.7. Library Preparation and Sequence Amplification

Library preparation was performed with a customized dual index version of Illumina’s Nextera XT protocol. The V3-V4 region of the 16S ribosomal subunit was amplified with custom polymerase chain reaction (PCR) primers and sequenced on an Illumina MiSeq.

### 2.8. Data Pre-Processing

Trimmomatic was used to remove sequencing adaptors, and low-quality reads [23]. The FLASH algorithm was used to read merging and automated rejection of low-quality sequences [24]. Quality screening for length and ambiguous bases was performed with proprietary scripts [23].

### 2.9. Data Analysis

**Figure 1:**
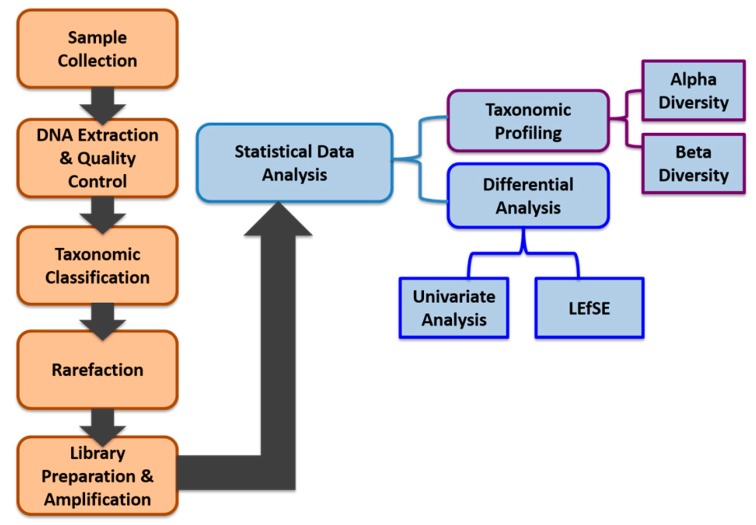
Workflow for the salivary microbiome data generation, pre-processing, and analysis.

We applied a comprehensive bioinformatics analysis approach integrating both robust exploratory data analysis and visualization methods focusing on taxonomic profiling, combined with standard statistical differential analysis approaches such as univariate analysis methods to identify statistically significant features in terms of their abundance between different smokers and non-smokers. We also applied the linear discriminant analysis (LDA) effect size (LEfSe) method to support high-dimensional class comparisons. Our data generation, data preprocessing, and data analysis workflow is shown in Figure 1.

#### 2.9.1. Alpha (α) Diversity and Beta (β) Diversity

Taxonomic profiling: exploratory data analysis and visualization consisted of two main methods: (a) alpha diversity analysis for assessing diversity within a bacterial community or sample and (b) beta diversity analysis for determining the differences between microbial communities (i.e., between samples). Three different alpha diversity metrics (Shannon Index, Observed OTUs, Chao1 diversity) were calculated on rarefied OTU tables using the *alpha_rarefaction.py* workflow in QIIME 1.9.1 [25] and the results were determined by using Analysis of Variance for each alpha diversity metric. Tukey’s honestly significant difference (HSD) was applied to the AOV for analysis of variance (ANOVA), it is an R function to determine group-to-group comparisons. The R version 3.3.2 (*R Core Team*, *2015*) was used to perform the statistical analyses of alpha and beta diversity. Additionally, three beta metrics were used (Bray-Curtis, Weighted UniFrac, and Unweighted UniFrac) on the rarefied OTU table using the *beta_diversity.py* workflow in QIIME 1.9.1. Bray-Curtis dissimilarity was calculated on a species-level summarization of the rarefied OTU table [26]. Principal Coordinates Analysis (PCoA) was applied to each beta diversity distance matrix, using the *dudi.pco* function from the R *made4* package (version 1.48.0). The first two principal coordinates explaining the majority of the difference in data were plotted using R’s *ggplot2* package, version 2.2.1), with the indicated percentage of variance by each axis explained.

#### 2.9.2. Univariate Analysis

Two standard univariate tests, implemented in MicrobiomeAnalyst [22] from the Xia lab at McGill University in Canada, were applied to test for statistically significant abundant taxa between smokers and non-smokers. The tests were: (a) non-parametric Mann-Whitney test and (b) parametric t-test/ANOVA. Our differential analysis helps in identifying biologically or biochemically meaningful relationships or associations between taxa or features. The analyses were conducted at phylum and genus levels.

#### 2.9.3. LDA Effect Size (LEfSe)

This method is specifically designed for biomarker discovery and explanation in high-dimensional metagenomic data [27]. It incorporates statistical significance with biological consistency (effect size) estimation. It performs a non-parametric factorial Kruskal-Wallis (KW) sum-rank test to identify features with significant differential abundance with regard to experimental factor or class of interest, followed by Linear Discriminant Analysis (LDA) to calculate the effect size of each differentially abundant features. The result consists of all the features, the logarithmic value of the maximum mean among all the groups or classes, and if the features are differentially significant, the group with the highest mean and the logarithmic LDA score (Effect Size). Features are considered to be significant based on their adjusted *p*-values (i.e., false discovery rate (FDR) values), applying an adjusted *p*-value cutoff = 0.05.

## 3. Results

### 3.1. Demographic Data of the Study Subjects

One hundred subjects were recruited in this study to attain salivary specimens (57 males and 43 females; 51 non-smokers and 49 smokers) to perform 16S rRNA gene sequencing. A total of 1308 OTUs were identified within the salivary microbiota of the 100 subjects who participated in this study. A designed questionnaire (Table 1) was developed to collect demographic data in addition to smoking history. As in Table 1, the smoking status of 100 subjects of the study summarized as the following; 23% female non-smokers, 28% male non-smokers, 20% female smokers, and 29% male smokers. Other descriptive characteristics in Table 1 included data with more than 50% between 21–25 years of age, ethnicity (95% white), education (76% bachelor degree), smoking duration (20% from 6–10 years), number of cigarette (20% from 10–20 cigarettes), and teeth brushing (89% brushing teeth). The difference in age was statistically significant at *p*-value ≤ 0.05. No statistically significant difference in the number of smokers versus non-smokers was observed at *p*-value = 0.05.

### 3.2. Alpha (α) Diversity Metrics

**Figure 2:**
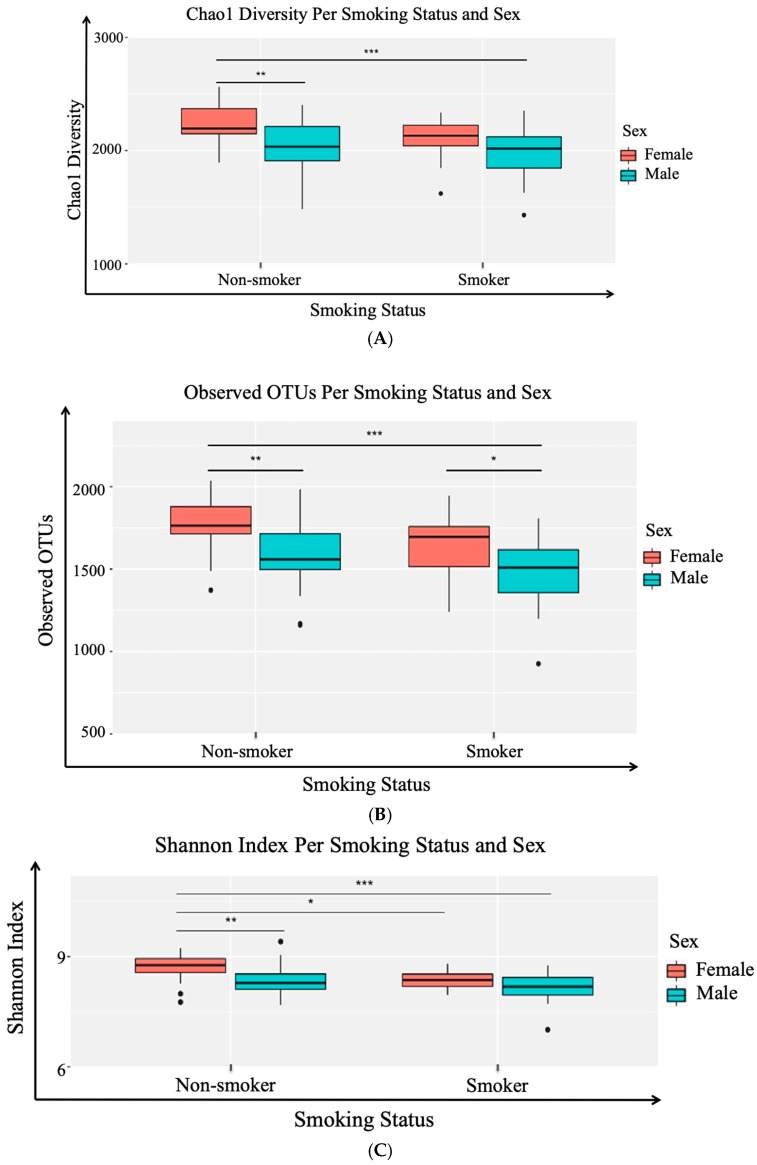
Boxplots of the three studied alpha diversity metrics: (**A**) Chao1 (community richness), (**B**) observed OTUs (community uniqueness), and (**C**) Shannon (community evenness or entropy). Red boxplots represent females and green boxplots represent males. Statistically significant differences were determined using analysis of variance with alpha diversity as the response variable, and smoking status and sex as crossed predictor variables, with Tukey’s HSD for group-specific differences. * Statistically significant at adjusted (*p*-value < 0.05), ** Statistically significant at adjusted (*p*-value < 0.01) and *** Statistically significant at adjusted (*p*-value < 0.001).

To understand the variations in the salivary microbiota, alpha diversity metrics were calculated on rarefied OTU tables for comparison groups based on gender and smoking status. The α metrics included Chao1 (community richness), Observed OTUs (community uniqueness), and Shannon (community evenness or entropy) within each comparison group (male smokers, female smokers, male non-smokers, and female non-smoker). Statistically significant differences at adjusted *p*-value < 0.05 were determined using analysis of variance with alpha diversity as the response variable on the *y*-axis, and smoking and gender status as crossed predictor variables on the *x*-axis (Figure 2). The Chao1 metric analysis showed statistically significant higher richness in smokers versus non-smokers, and interestingly, a statistically significant higher richness among female non-smokers versus male non-smokers (Figure 2A). The metrics of observed OTUs represented the amount of unique OTUs found in each sample to measure the diversity within samples in an ecosystem. Relatively, there was a significant community uniqueness in non-smokers versus non-smokers, in addition to a considerable community uniqueness in females versus males regardless of the smoking status (Figure 2B). In the last calculated α metric, Shannon metric considers the number and abundance of OTUs found in a sample together; the more evenly abundant the OTUs present in a sample, the higher the Shannon index. Shannon’s analysis showed that smokers and non-smokers were evenly abundant (Figure 2C).

### 3.3. Beta (β) Diversity Metrics

**Figure 3:**
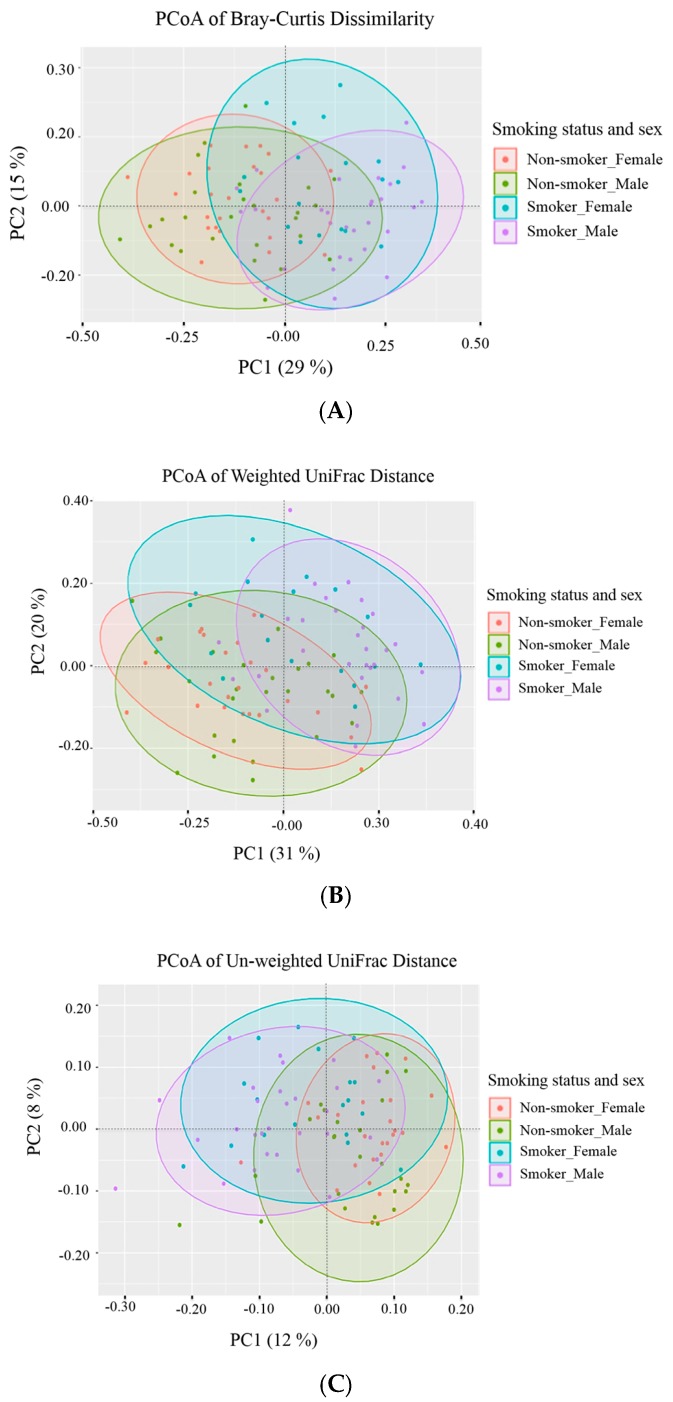
Principal Coordinates Analysis (PCoA) of the distance matrix generated using three distance metrics: (**A**) Bray-Curtis dissimilarity data, (**B**) Weighted UniFrac, and (**C**) Unweighted UniFrac. The x and y axes correspond to the first and second major principal coordinates (PC1 and PC2) identified from the PCoA analysis. Each principal coordinate explains a certain fraction of the variability (indicated by the percentage between brackets on each axis) observed in the data set. The principal coordinates PC1 and PC2 are plotted to create a visual two-dimensional (2D) representation of the multidimensional microbial community compositional differences between tested samples. Each sample is represented by a point and colored based on the smoking status and the sex of tested human subjects: Female non-smokers (red), male non-smokers (green), female smokers (teal green), and male smokers (magenta). The distance between the points represents the similarity of those samples (closer together = more similar).

Principal Coordinates Analysis (PCoA) is a tool used to visualize the profiling of sample clustering based on the similarity to each other, and this helps determine whether changes identified during beta diversity analysis are directed changes or random noise. Plots for distance matrices were generated using three beta diversity metrics, Bray-Curtis, Weighted UniFrac, and Unweighted UniFrac, to highlight the separation of samples per group. Bray-Curtis compositional dissimilarity metric compared the abundance of each OTU between two samples to give a parameter between 0 and 1. This absolute metric quantified the difference in abundance between two communities and was useful for identifying how two microbiome samples are similar. Weighted (quantitative) UniFrac distance is a dissimilarity metric that uses the phylogenetic distribution and the abundance of the OTUs in a sample to calculate the distance between two samples. The unweighted (qualitative) UniFrac distance measures the phylogenetic distribution of the OTUs in a sample but relies on the presence or absence of OTUs rather than their abundance. The results of beta diversity are in Table 2. The Bray-Curtis analysis in Figure 3A showed that there are significant compositional dissimilarities among the different comparison groups, except for the male non-smokers versus female non-smokers. When considering the weighted UniFrac, results in Figure 3B showed significant compositional dissimilarities among the different comparison groups except for the male non-smokers versus female non-smokers and male smokers versus female smokers. When considering the Unweighted UniFrac, results in Figure 3C showed significant compositional dissimilarities among the different comparison groups except for male smokers versus female smokers. In general, most of bacterial OTUs were shared all-over the groups.

All performed group comparisons of β-diversity were assessed with a pairwise permutational multivariate ANOVA (PerMANOVA) using the ‘pairwise.adonis’ function from the vegan R package, which can be found here: https://github.com/pmartinezarbizu/pairwiseAdonis. The pairwise function takes each pair and runs the adonis test independently. Then, it applies a Bonferonni correction with the ‘p.adjust’ function which divides the original α-values by the number of comparisons performed. In this study, we performed the following six comparisons: Nonsmoker_male versus smoker_male, nonsmoker_male versus nonsmoker_female, nonsmoker_male versus smoker_female, smoker_male versus nonsmoker_female, smoker_male versus smoker_female, and nonsmoker_female versus smoker_female. Therefore, the original *p*-values (*p*-value) were multiplied by 6 to get the Bonferroni-adjusted *p*-values (*p*-adj) reported in Table 2.

### 3.4. Visualization of the Taxonomic Profiling Results

**Figure 4:**
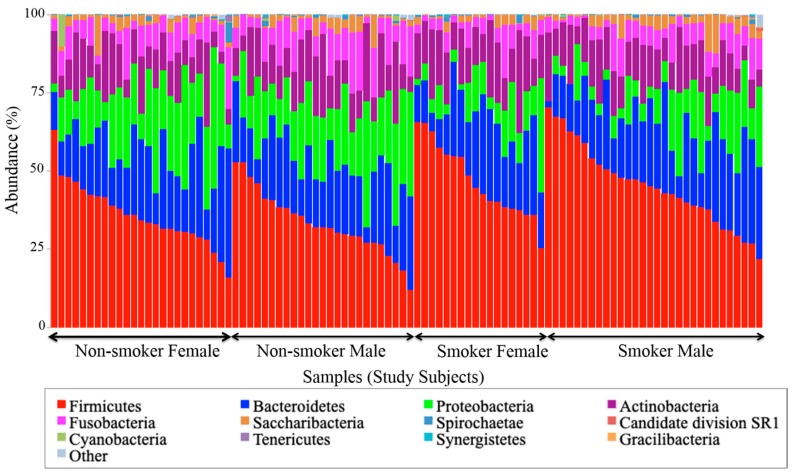
Taxonomic composition represented by the abundance (percentage) of phyla per smoking status per gender for each sample in each comparison group.

**Figure 5:**
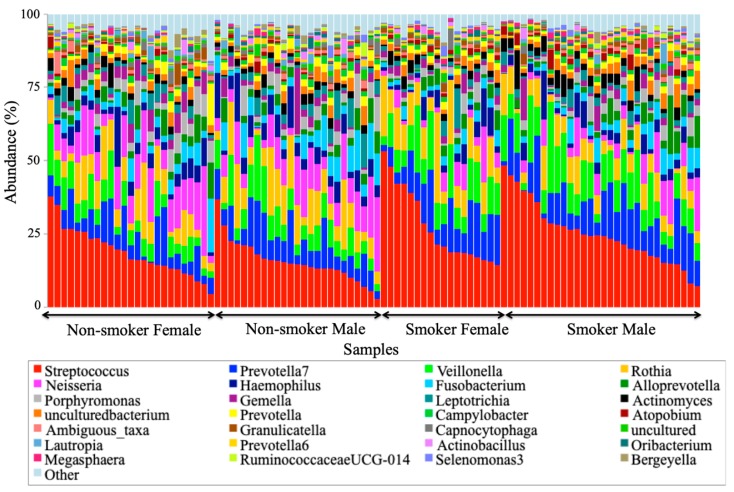
The relative abundance of genera per sample group per smoking status and gender.

Stacked bar charts (precisely, 100% stacked) were used for the visualization of the taxonomic profiling results generated from abundance data in OTU tables. These charts enabled the display of the composition and the distribution of most abundant bacterial taxa at the phyla (Figure 4) and genera (Figure 5) levels across 100 studied salivary samples from smokers and non-smokers. Two samples were dropped out of the analysis because of some missing demographic data about them to avoid any type of uncertainty errors. Both Figure 4 and Figure 5 clearly show that the taxonomic identity and distribution of the salivary microbiota were conserved among all tested samples from smoker and non-smoker human subjects at phyla and genera levels. At the phylum level, our results revealed that that the salivary microbiome was largely predominated by *Firmicutes*, *Bacteriodetes*, *Proteobacteria*, *Actinobacteria*, *Fusobacteria*, and *Saccharibacteria.*

### 3.5. Univariate Analysis

To test if there are any statistically significant differences in the abundance of abundant taxa between smokers and non-smokers, we performed both parametric and non-parametric univariate analyses. Non-parametric univariate analyses relied on Mann–Whitney and/or Kruskal-Wallis (which is an extension to Mann–Whitney test to test for more than two samples) tests. Our results revealed that three abundant phyla including *Proteobacteria*, *Firmicutes*, and *Fusobacteria*, showed statistically significant differences in abundance between smokers and non-smokers. The false discovery rate (FDR) values reported in Table 3 were used to assess statistical significance of the univariate analysis results setting the cutoff at 0.05.

Results of the univariate analyses are reported in Table 3 and Table 4 for phyla and genera levels subsequently. Features were considered statistically significant based on their FDRs with a significance level set at 0.05. Table 3 showed the phylum *Firmicutes* is significantly more abundant in smokers versus non-smokers, while *Proteobacteria* and *Fusobacteria* were less abundant in the non-smokers’ group.

**Figure 6:**
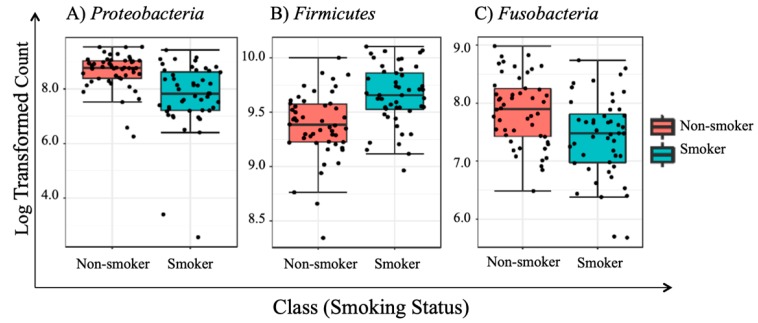
Boxplot for the three statistically impacted features identified by univariate analysis at the phylum level for non-smokers (red box) versus smokers (blue box) regardless of gender, statistically significant *p*-values with a significance level set at 0.05.

At the genera level, six specific genera consisting of Streptococcus, Prevotella, Vellionella, Rothia, Neisseria, and Haemophilus, dominated the salivary microbiota of all examined samples from both smokers and non-smokers (Figure 5). Univariate analysis results (Table 4) showed that Streptococcus, Prevotella, and Veillonella were significantly more prevalent in smokers than in non-smokers, whereas Neisseria, a bacterial genus that is part of the healthy flora in the human oral cavity, was significantly lower in smokers versus non-smokers (Figure 6).

### 3.6. LDA Effect Size (LEfSe)

**Figure 7:**
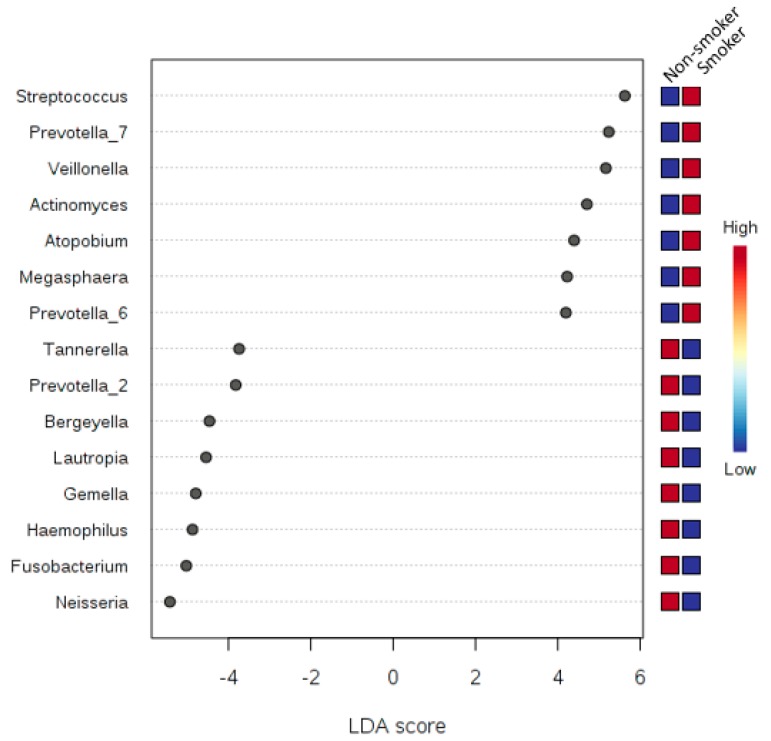
A plot of the LDA scores of the top 15 genera showing statistically significant differences between smokers and non-smokers. LDA scores on the *x*-axis and genera on the *y*-axis. The color-coding in the squares of the right side of the plot refers to the cumulative abundance of each genus in each binned group, where red means high cumulative abundance and blue means low cumulative abundance.

**Figure 8:**
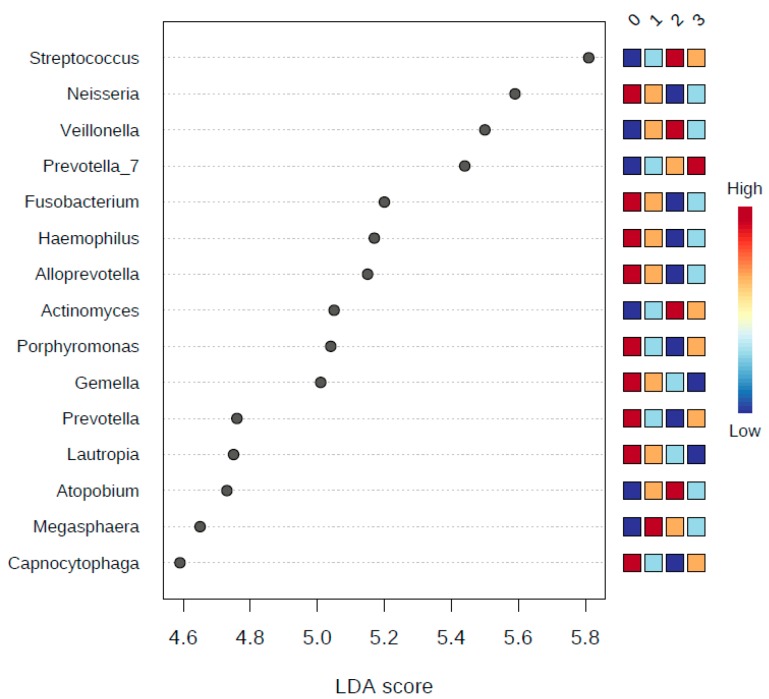
A plot of the LDA scores of the top 15 genera showing statistically significant differences between binned years of smoking. LDA scores on the *x*-axis, and genera on the *y*-axis. The color-coding in the squares of the right side of the plot refers to the cumulative abundance of each genus in each binned group, where red means high cumulative abundance and blue means low cumulative abundance.

**Figure 9:**
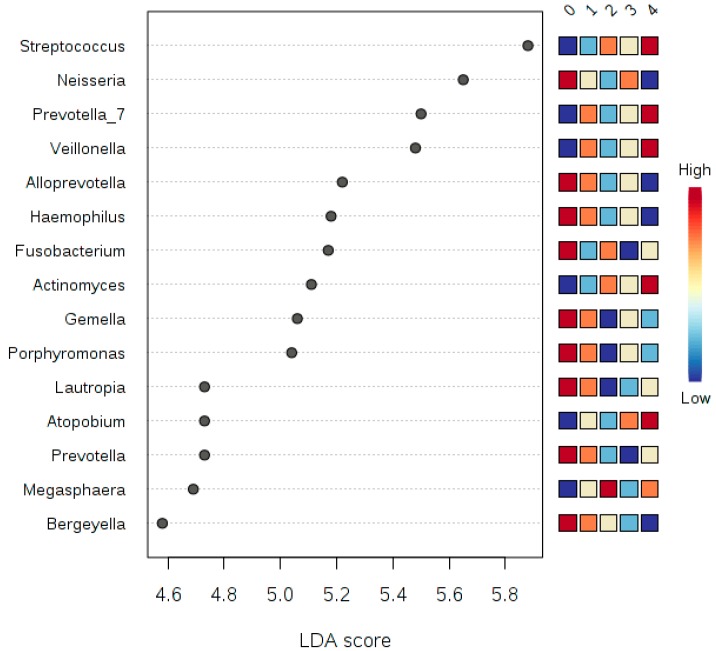
A plot of the LDA scores of the top 15 genera showing statistically significant differences between binned numbers of cigarettes smoked by human subjects. LDA scores are on the *x*-axis and genera on the *y*-axis. The color-coding in the squares of the right side of the plot refers to the cumulative abundance of each genus in each binned group, where red means high cumulative abundance and blue means low cumulative abundance.

Correlation analysis using LDA Effect Size (LEfSe) was performed at the phyla and genera taxonomy levels, and testing for five experimental factors, namely: class (smoker or non-smoker), sex (female or male), the number of smoking years, the number of cigarettes smoked (Figure 7, Figure 8 and Figure 9) and teeth-brushing habits (brushing or no brushing). To get meaningful insight from the number of years of smoking and the number of cigarettes smoked, we binned all numbers into four and five classes subsequently (Table 5). For the number of smoking years we binned the numbers into four classes: class 0 for zero years of smoking (i.e., son-smoker), class 1 for smoking years from 1 to less than 5, class 2 for smoking years from 5 to less than 10, and class 3 for more than 10 years. For the number of cigarettes smoked per day, we binned the numbers into five classes: Class 0 for zero number of cigarettes, class 1 for a number of cigarettes from 1 to less than 10, class 2 from 10 to less than 20, class 3 from 20 to less than 30, class 4 more than 30 cigarettes per day. We also applied a *t*-test/ANOVA to study the effect of teeth brushing on the salivary microbiome (not tabulated). We studied the effects at the phyla and genera levels and identified the phylum *Synergistetes* (*p*-value = 0.0015, and FDR = 0.0133) as a statistically significant phylum distinguishing the human subjects that brush and those that do not brush their teeth. At the genus level however, we found there are five genera having to show statistically significant differences in abundance between smokers and non-smokers. The five genera were: (1) Haemophilus (*p*-value = 2.2273 × 10^−4^ and FDR = 0.0156), (2) Filifactor (*p*-value = 3.8769 × 10^−4^ and FDR = 0.0204), (3) Eubacterium_brachy_group (*p*-value = 5.818 × 10^−4^ and FDR = 0.0204), (4) Fretibacterium (*p*-value = 0.0015 and FDR = 0.0346), and (5) Parvimonas (*p*-value = 0.0027 and FDR = 0.047327).

### 3.7. Multivariate Analysis by Linear Models (MaAsLin)

In order to identify (or rule out the presence of) any confounding covariates that may contribute to the observed changes in microbiome composition with smoking status, we applied a multivariate statistical approach from MaAsLin [28] to relate smoking status to microbiome structure and function while accounting for potential correlates and confounding factors such as tooth-brushing and gender. Clades were tested for statistically significant associations with demographic metadata of interest by using a novel multivariate algorithm. Each clade was normalized with a variance-stabilizing arcsine square-root transformation and evaluated with a general linear model using the glm package in R. Model selection for sparse data was performed per clade using boosting from gbm package. A multivariate linear model associating all available metadata with each clade independently was boosted, and any metadata selected in at least 1% of these iterations was finally tested for significance in a standard generalized linear model. Within each metadatum/clade association independently, multiple comparisons over factor levels were adjusted using a Bonferonni correction; multiple hypothesis tests over all clades and metadata were adjusted to produce a final Benjamini and Hochberg false discovery rate (i.e., *Q*-value). Significant association was considered below a q-value threshold of 0.25. MaAsLin Analysis did not result in any significant confounding variables that could explain the differences in microbiome composition, on the phyla and genera levels, between smokers and non-smokers. These results confirmed the that microbiome compositional differences between human subjects are attributed to the smoking status identified by univariate analyses.

## 4. Discussion

To the best of our knowledge, this paper represents a first of its kind report in Jordan, documenting statistically significant changes in the salivary human microbiome composition between smoker and non-smoker human subjects. Our methods relied on high throughput next-generation sequencing of the 16S rRNA marker gene determined in unstimulated salivary samples. Based on the outcomes of previous studies addressing the adverse effects of smoking on health in general, it was anticipated that pathogenic bacteria might be present at the expense of the commensal flora in smokers. This study showed that alpha and beta diversity displayed intra and inter-individual variations. However, the profile clustering direction for each study group (male smokers, female smokers, male non-smokers, and female non-smoker) was apparent with interesting overlapping Venn diagrams for male and female non-smokers versus male and female smokers. This implies a significant response to smoking regardless of gender, even with slight significant statistical variation between males and females in general. *Firmicutes*, *Proteobacteria*, and *Bacteroidetes* were found to have the highest relative abundance percentage of the community at phylum level in all samples. However, smoking had affected the *Firmicutes*, *Proteobacteria*, and *Fusobacteria*, as *Firmicutes* was statistically elevated in smokers at the expense of *Proteobacteria* and *Fusobacteria* in non-smokers. This implies that smoking has a critical impact on the homeostasis of human salivary microbiome. The biological meaning of these findings was not evident until we performed the analysis at genera level.

At the genus level, *Streptococcus*, *Prevotella*, *Vellionella*, *Rothia*, *Neisseria*, and *Haemophilus* predominated the salivary microbiota of all examined samples. *Streptococcus*, *Prevotella*, and *Veillonella* were the most significantly predominant genera among smokers at the expense of *Neisseria* that are healthy flora in the human oral cavity, which has been significantly decreased among smokers. The increased levels of *Streptococcus* and *Veillonella* and the reduced level of *Neisseria* were consistent with an extensive study of in a thorough survey of cigarette smoking and oral microbiome among American adults [8]. The reduced level of anaerobic *Neisseria* in this study is consistent with a human oral microbiota study [29], which might be related to the effect of oxygen deprivation in the oral cavity caused by smoking. The predominance of the anaerobic *Veillonella* and the facultative anaerobic *Streptococcus* may explain their success in tolerating the lack of oxygen in the smoking microenvironment. Elevated *Prevotella* was correlated to oral malodor (halitosis) [30], which can be caused by smoking in this study, which is consistent with a clinical review by Porter and Scully [31].

Since statistically significant taxa do not always convey the biological messages, we want to arrive in to make important discoveries later on. We performed additional statistical tests to build confidence in the prioritized taxa and make sure that these taxa are able to explain the differences between the studies classes of smokers and non-smokers. This, in addition to a subsequent related classification based on the number of years of smoking, the number of cigarettes smoked, and whether the human subjects brush or did not brush their teeth, in terms of teeth brushing the phylum *Synergistetes* were identified as a statistically significant phylum distinguishing the human subjects that brush and those that do not brush their teeth. *Synergistetes* has been reported in both periodontal health and disease [32]; thus, a further investigation at the species level of *Synergistetes* is needed. Our tests relied on LEfSe, which combines standard tests for statistical significance with additional tests encoding biological consistency and effect relevance. Based upon our LEfSe results analysis in Figure 7, Figure 8 and Figure 9, we can see that there is a microbial signature distinguishing smokers from non-smokers, which is consistent with our univariate analysis except that the abundance of *candidate division SR1* was statistically significant (Table 5). The *candidate division SR1* usually described in the literature as unknown or unaffiliated [33]. We did not see such a clear signature distinguishing the different classes of human subjects resulting from the binned numbers of years of smoking and the binned numbers of the number of cigarettes smoked.

We can suggest that there is microbial signature at the genera level can be used to classify smokers and non-smokers by LEfSe based on the salivary abundance of the 15 genera including, but not limited to, *Streptococcus*, *Prevotella*, and *Veillonella*, which are all more abundance in smokers relative to non-smokers, and *Neisseria,* which is more abundant in non-smokers relative to smokers.

It is worthy to note that infections are believed to be a cause of carcinogenesis, alongside other known risk factors such as smoking tobacco and consuming alcohol. The case for role infections in carcinogenesis is increasingly solidified with evidence that the inflammation bacteria can secrete endotoxins, which in turn might induce DNA damage in mouth epithelial tissue [34,35]. A positive correlation between proinflammatory cytokine levels and commensal bacteria was observed in smokers, but that correlation was not present for non-smokers. A previous study suggested that smoking affects both the composition of the nascent biofilm and the host reaction to this colonization [3]. The elevated abundance of *Streptococcus*, *Prevotella*, and *Veillonella* in this study should be considered in future research to explore the feasibility of being a salivary diagnostic predictor in Jordanian smokers for oral squamous cell carcinomas. For example, a previous report concluded that some specific taxa have a significant correlation with epithelial precursor regions and oral cancer, taxa such as *Streptococcus* spp., *Veillonella*, *Porphyromonas*, *Fusobacterium*, *Prevotella*, *Actinomyces*, *Clostridium*, *Haemophilus*, and *Enterobacteriaceae* [36].

Although we were successful in generating high-quality sequencing data that enabled the subsequent bioinformatics analysis to identify significant compositional differences in the salivary microbiome between smokers and non-smokers, this study has some limitations that we should disclose here. First, targeting only two hypervariable regions, V3-V4 on the 16S rRNA gene, might group closely related taxa into a single taxonomic unit. However, there is sufficient evidence in the biomedical literature indicating that the V3-V4 exploration is adequate to produce a reliable phylogenetic ranking at phyla and genus levels, but usually not at species level. Second, even though 16S rRNA remains the most efficient available approach to study microbial communities, it suffers mosaicism, intra-genomic heterogeneity, and lacks a universal threshold of what is known as sequence identity value [37]. Third, we could not control for other confounders for example lifestyle, exact and complete oral health beyond yes or no teeth brushing, alcohol consumption, drug abuse, and chemical and physical properties of saliva. Last, it is challenging to determine whether the existing microbial colonization is a long-term one or not.

## 5. Conclusions

The present investigation systematically combined NGS and bioinformatics to examine the effect of tobacco smoking on the unstimulated salivary microbiome in human subjects by targeting the 16S rRNA gene. The results of this investigation confirmed a proven impact of smoking on the core salivary microbiome between smokers and non-smokers in terms of relative abundance percentages. Six genera—*Streptococcus*, *Prevotella*, *Vellionella*, *Rothia*, *Neisseria*, and *Haemophilus*—predominated the salivary microbiota of all samples with different proportions. Three genera—*Streptococcus*, *Prevotella*, and *Veillonella*—showed significant differences between the comparison groups at the expense of *Neisseria*. Smoking has a definite impact on shifting the salivary microbiota in smokers. Further studies are needed to explore if dominant genera can be utilized as diagnostic biomarkers to predict or early detect the periodontitis and oral epithelial precursor lesions among smokers in Jordan. Herein, proteomics and metabolomics studies are recommended to fully understand the xenobiotic metabolism and its effect on the interrelationships among bacteria of the salivary microbiome and how they affect the growth of each other.

## Figures and Tables

**Table 1:** Demographic data of the study population.

Gender (n ^a^)	Age in Years (mean ± SD)	Ethnicity	Education	Tobacco Smokers versus Nonsmokers (n)	Smoking Duration (n)	Number of Cigarettes (n)	Teeth Brushing
Male (57)	23.9 ± 6.20	White (95)	School (20)	Smokers (49)	<1 Year (2)	1–10 (15)	Brush (89)
Female (43)	27.1 ± 7.57	Black (4)	Bachelor (76)	Non-smokers (51)	1–5 Years (15)	10–20 (20)	Do not Brush (9)
		Caucasian (1)	Master (4)	Male non-smokers (28)	6–10 Years (20)	21–30 (6)	
				Female non-smokers (23)	>10 Years (9)	>30 (5)	
				Male smokers (29)			
				Female smokers (20)			
***p*** **-value**	0.0245 * (age)			0.6693 (smokers versus non-smokers)			

n ^a^ is the number of samples—the asterisks (*) indicate a statistically significant *p*-value < 0.05. Note: non-smokers are defined as subjects who never smoked before.

**Table 2:** The Principal Coordinates Analysis (PCoA) plots of the three beta diversity metrics were used (Bray-Curtis, Weighted UniFrac, and Unweighted UniFrac) measuring significant differences in beta diversity.

Group	Bray-Curtis Dissimilarity		Weighted UniFrac		Unweighted UniFrac	
	*p*-Value	*p*-Adjusted	*p*-Value	*p*-Adjusted	*p*-Value	*p*-Adjusted
Male non-smokers versus male smokers	0.001	0.006 *	0.001	0.006 *	0.001	0.006 *
Male non-smokers versus female non-smokers	0.074	0.444	0.171	1.000	0.003	0.018 *
Male non-smokers versus female smokers	0.001	0.006 *	0.001	0.006 *	0.001	0.006 *
Male smokers versus female non-smokers	0.001	0.006 *	0.001	0.006 *	0.001	0.006 *
Male smokers versus female smokers	0.005	0.030 *	0.013	0.078	0.020	0.120
Female non-smokers versus female smokers	0.001	0.006 *	0.001	0.006 *	0.001	0.006 *

* Statistically significant at Bonferroni adjusted *p*-value significance level set at 0.05.

**Table 3:** The top features identified by univariate analysis at the phylum level for non-smokers versus smokers (regardless of gender).

#	Phylum	*p*-Values	FDR
*1*	***Proteobacteria***	2.80 × 10^−6^ *	2.52 × 10^−5^ *
*2*	***Firmicutes***	7.16 × 10^−6^ *	3.22 × 10^−5^ *
*3*	***Fusobacteria***	0.0017 *	0.0053 *
*4*	*Spirochaetae*	0.2924	0.6579
*5*	*Synergistetes*	0.4506	0.8112
*6*	*Actinobacteria*	0.5616	0.8424
*7*	*Candidate_division_SR1*	0.7550	0.8939
*8*	*Saccharibacteria*	0.7946	0.8939
*9*	*Bacteroidetes*	0.9579	0.9579

The asterisks (*) and bold names indicate statistically significant *p*-values with a significance level set at 0.05. Note: when the asterisk is on the right side of the value, it means significantly higher among smokers; when it is on the left side of the value, it means significantly higher among non-smokers.

**Table 4:** The critical features identified by Univariate analysis at the Genus level.

#	Genus	*p*-Values	FDR
*1*	***Neisseria***	4.58 × 10^−6^ *	0.0003 *
*2*	***Streptococcus***	3.27 × 10^−5^ *	0.0011 *
*3*	***Prevotella***	0.0013 *	0.0299 *
*4*	***Veillonella***	0.0016 *	0.0299 *
*5*	*Bergeyella*	0.0043	0.0522
*6*	*Eikenella*	0.0049	0.0522
*7*	*Johnsonella*	0.0050	0.0522
*8*	*Fusobacterium*	0.0062	0.0566
*9*	*Megasphaera*	0.0076	0.0591

The asterisks (*) and bold names indicate statistically significant *p*-values with a significance level set at 0.05. Note: When the asterisk is on the right side of the value, it means significantly higher among smokers; when it is on the left side of the value, it means significantly higher among non-smokers.

**Table 5:** Important taxonomic features at the phylum level identified by LEfSe.

**Smoker versus Non-Smoker**								
**Phylum**	*p*-Value *	**FDR**	**Abundance**					**LDA Score**
			**Non-Smoker**	**Smoker**				
Proteobacteria	**6.06 × 10^−6^**	**5.45 × 10^−5^**	1,828,000	1,003,200				−5.62
Firmicutes	**9.99 × 10^−5^**	**4.50 × 10^−4^**	3,539,800	4,517,700				5.69
Fusobacteria	**2.54 × 10^−3^**	**7.62 × 10^−3^**	882,530	583,700				−5.17
Candidate_division_SR1	7.27 × 10^−2^	1.64 × 10^−1^	21,233	18,901				−3.07
Synergistetes	1.27 × 10^−1^	2.28 × 10^−1^	1706.6	1317.4				−2.29
Actinobacteria	2.73 × 10^−1^	4.10 × 10^−1^	1,334,300	1,422,600				4.65
Spirochaetae	3.87 × 10^−1^	4.97 × 10^−1^	13,132	6343.5				−3.53
Saccharibacteria	5.71 × 10^−1^	6.42 × 10^−1^	265,320	288,340				4.06
Bacteroidetes	6.44 × 10^−1^	6.44 × 10^−1^	2,114,000	2,157,900				4.34
**Binned Number of Years of Smoking**								
**Phylum**	*p*-value *	**FDR**	**Abundance**					**LDA Score**
			**Class 0**	**Class 1**	**Class 2**	**Class 3**		
Proteobacteria	**1.11 × 10^−9^**	**9.99 × 10^−9^**	1,968,900	1,072,400	619,710	830,020		5.83
Firmicutes	**2.26 × 10^−7^**	**1.02 × 10^−6^**	3,368,600	4,624,900	5,071,300	4,371,100		5.93
Fusobacteria	**4.06 × 10^−4^**	**1.15 × 10^−3^**	910,010	652,060	500,500	487,050		5.33
Candidate_division_SR1	**5.10 × 10^−4^**	**1.15 × 10^−3^**	32,448	2398.8	8833.8	5397.3		4.18
Actinobacteria	4.17 × 10^−2^	7.51 × 10^−2^	1,244,500	1,287,900	1,664,300	1,606,300		5.32
Synergistetes	2.12 × 10^−1^	3.17 × 10^−1^	1579.1	538.51	799.61	3512.6		3.17
Spirochaetae	2.55 × 10^−1^	3.27 × 10^−1^	13,425	4161.2	5597.2	8196		3.67
Bacteroidetes	4.70 × 10^−1^	5.28 × 10^−1^	2,203,300	2,055,600	1,867,200	2,337,300		5.37
Saccharibacteria	8.80 × 10^−1^	8.80 × 10^−1^	257,200	300,020	261,740	351,060		4.67
**Binned Number of Cigarettes**								
**Phylum**	*p*-value *	**FDR**	**Abundance**					**LDA Score**
			**Class 0**	**Class 1**	**Class 2**	**Class 3**	**Class 4**	
Proteobacteria	**5.63 × 10^−9^**	**5.06 × 10^−8^**	1,968,900	1,096,000	799,060	865,290	545,000	5.85
Firmicutes	**4.48 × 10^−7^**	**2.01 × 10^−6^**	3,368,600	4,546,800	5,000,800	4,423,500	4,993,400	5.91
Fusobacteria	**1.53 × 10^−3^**	**4.60 × 10^−3^**	910,010	538,570	647,930	492,040	505,900	5.32
Candidate_division_SR1	**4.04 × 10^−3^**	**9.10 × 10^−3^**	32,448	5620.1	5607.6	7110.8	4922.3	4.14
Actinobacteria	6.14 × 10^−2^	1.10 × 10^−1^	1,244,500	1,279,700	1,438,900	1,882,600	1,458,900	5.50
Spirochaetae	8.35 × 10^−2^	1.25 × 10^−1^	13,425	4146.5	4018.8	4911.9	10,250	3.67
Bacteroidetes	3.95 × 10^−1^	5.08 × 10^−1^	2,203,300	2,298,200	1,761,300	1,958,300	2,234,600	5.43
Synergistetes	9.04 × 10^−1^	9.97 × 10^−1^	1579.1	856.72	778.84	885.28	3302.3	3.10
Saccharibacteria	9.97 × 10^−1^	9.97 × 10^−1^	257,200	230,080	341,600	365,390	243,750	4.83

* *p*-values were based on the FDR test, values with a significance level set at 0.05 are in bold.

## Data Privacy and Availability Statement

The Council of Scientific Research at the German Jordanian University restricts the raw data used to support the findings of this study to protect human subjects’ privacy. Data are available from Walid Al-Zyoud [walid.alzyoud@gju.edu.jo] for researchers who meet the criteria for access to confidential data.

## Appendix A. Sequencing Quality Metrics

A total number of 25,000 sequences were retrieved and passed the quality test (Table A1). As an evaluation of the overall quality of a sequencing run, the % ≥ Q30 score was 79.62%, which is the proportion of base calls that have a confidence score of 30 or more. The cluster density in the sequencing run was 914 ± 16 k/mm^2^ that exceeded the minimum requirement ≥ 800 k/mm^2^, which gives an idea on how efficiently the sequencer can bind sequences of DNA to the flow cell. The clusters passing filter percentage was 91.47 ± 0.75% that exceeded the minimum requirement ≥ 80%, which is the proportion of clusters that meet the sequencer’s minimum quality threshold for sequence quality. Only clusters that passed filter were included in the sequencer’s FASTQ output, which is a text-based format for storing nucleotide sequences and its quality scores. The sequencing yield was 12.36 Gbp, which refers to how many nucleotide base pairs were called by the sequencer. 1 Gbp (Giga base pair) means the sequencer generated 1 billion base pairs of output; the minimum requirement is ≥13.2 Gbp. The PhiX alignment was 19.01%; the PhiX is a sequencing library that was used as a positive control of each sequencing run. The sequencer aligns to the PhiX library to calculate sequence-based quality control metrics. We require that the percentage of reads that align to the PhiX library is within 20% of the spike-in amount of PhiX.

**Table A1:** Sequence quality metrics per sequencing run.

Metric	Minimum Requirement	Results
% ≥ Q30	≥70%	79.62%
Cluster Density	≥800 k/mm^2^	914 ± 16 k/mm^2^
Clusters Passing Filter (%)	≥80%	91.47 ± 0.75%
Sequencing Yield	≥13.2 Gbp	12.36 Gbp
PhiX Alignment (%)	12%–18%	19.01%

All the classified sequences that were used here passed the threshold that is represented by the dashed line in Figure A1.
